# Blocking *Plasmodium* Development in Mosquitoes: A Powerful New Approach for Expanding Malaria Control Efforts

**DOI:** 10.4269/ajtmh.19-0318

**Published:** 2019-07-01

**Authors:** Jeremy Burrows, David A. Fidock, Robert Scott Miller, Sarah Rees

**Affiliations:** 1 Medicines for Malaria Venture, Geneva, Switzerland;; 2 Department of Microbiology and Immunology and Division of Infectious Diseases, Department of Medicine, Columbia University Irving Medical Center, New York, New York;; 3 Bill & Melinda Gates Foundation, Seattle, Washington;; 4 Innovative Vector Control Consortium, Liverpool, United Kingdom

A recent article from Paton et al. “Exposing *Anopheles* mosquitoes to antimalarials blocks transmission of *Plasmodium* parasites”^1^ has deservedly drawn considerable interest. In this landmark study, the authors showed that adding atovaquone to a glass substrate on which blood-fed *Anopheles* mosquitoes rested led to killing of *Plasmodium falciparum* parasites resident in the midgut blood meal. The atovaquone concentrations required for effective killing were below those of permethrin, a potent neurotoxic insecticide used in long-lasting insecticide-treated bed-nets (LLINs). Modeling studies predicted that adding atovaquone to LLINs would substantially increase bed-net effectiveness across a broad range of transmission settings by reducing the prevalence of malarial infections. LLINs have been estimated to account for 68% of the reduction in numbers of malaria cases since 2,000, but their effectiveness is challenged by the rise of resistance to pyrethroid insecticides. A vital need for new malaria-prevention strategies is underscored by evidence that progress against malaria has plateaued in the past few years,^2^ with an estimated 435,000 deaths in 2017.

Insecticides have traditionally been delivered to adult mosquitoes via aerosol contact, ingestion of an “attractive toxic sugar bait,”^3^ or surface contact on a bed-net or a wall. The idea of delivering an antimalarial via surface contact with a mosquito seeking a blood meal is a truly innovative approach to disrupting the *Plasmodium* transmission cycle, and has many attractions. First, the technology and know-how to design and deliver compounds by this approach, optimized through the use of LLINs, are well established. Second, *Plasmodium* parasite numbers in the mosquito vector are low, with typically no more than five oocysts per midgut, inside which form several thousand motile sporozoites that are infectious for humans. By comparison, severely ill malaria patients can carry upward of 10^12^ asexual blood-stage parasites. The mosquito stages, therefore, carry a far lower risk, than blood stages, of generating resistance de novo (for atovaquone, *P. falciparum* resistance can be selected from ∼10^8^ asexual blood-stage parasites). Third, the potential impact of transmission blocking, as elegantly demonstrated in the article, can be substantial with the right compound and mode of action. Fourth, such an approach builds on and complements other existing interventions, and could attack the parasite through mechanisms not used in case management. These attributes would be unnecessary if bed-nets were impregnated with fully effective insecticides that decimate local mosquito populations and block transmission. Recent data, however, show that *Anopheles* resistance to pyrethroids is spreading across Africa.^4,5^

Despite the lower risk of de novo resistance selection targeting the numerical bottleneck of *Plasmodium* development in the mosquito midgut, the net as a delivery system exposes a sporontocidal drug to important risks. Contact exposure of mosquitoes may well be much less than the studied 6 minutes, and drug exposure on a net surface is likely to diminish over years of use. This would result in subinhibitory exposure, not unlike adding chloroquine to salt in early malaria control efforts in Brazil.^6^ Malaria control programs focus on minimizing the risk of treatment failure typically through the use of fixed-dose combinations in which component drugs have distinct resistance mechanisms.^7,8^ As a matter of caution, a drug used to treat or prevent malaria, or, indeed, any drug cross-resistant with such an agent, should ideally not be used in a transmission-blocking strategy administered directly to mosquitoes, for example, on a bed-net or attractive toxic sugar bait, as this strategy would risk losing the efficacy of essential life-saving medicines. As stated in Paton et al.,^1^ the use of atovaquone (a marketed antimalarial for both treatment and prophylaxis in combination with proguanil) was a proof of principle, and was not presented as a call to policy. Indeed, this novel approach to killing vector-stage parasites through direct mosquito exposure will ideally use new transmission-blocking drugs with modes of resistance that differ from those of approved products. The safest way to achieve this would be with a drug that is effective against *Plasmodium* sexual stages in the mosquito without exerting selective pressure on asexual blood-stage parasites. Several antimalarial drugs with sporontocidal activity have been developed, including atovaquone and, most recently, tafenoquine.^9,10^ The pathway for approval of novel tools that prevent transmission, however, is arduous, and particularly so for products that are solely measured by impact on an epidemiological outcome.^11^ The approach could be further challenged if an intervention that cured mosquitoes of parasites was seen to improve mosquito fitness or fecundity.^12^

Despite the challenges to developing bed-nets that deliver an anti-*Plasmodium* drug, the good news is 3-fold. First, there is a renewed investment in developing and delivering novel insecticides for nets and indoor residual spraying that effectively kill mosquitoes resistant to current agents.^13^ Second, new delivery systems, such as attractive toxic sugar baits, may allow more standard dosing and greater compound stability compared with that with a complex net matrix used over years.^14,15^ Third, existing high-throughput phenotypic or target-based screens^16–18^ could be adapted to identify sporontocidal antimalarials—compounds that would block transmission in mosquitoes. Were such agents to have different mechanisms of resistance from those of approved antimalarials and insecticides, then with careful optimization of potency, physical properties, metabolic stability, and safety—all with a focus on low cost—an appropriate mosquito-targeted transmission-blocking agent could be delivered that is tailored for use within traditional vector control strategies. The study by Paton et al.^1^ stimulates a powerful new approach^19^ to reducing the global burden of malaria and potentially other mosquito vector-borne diseases.

## References

[1] Paton DG Childs LM Itoe MA Holmdahl IE Buckee CO Catteruccia F, 2019 Exposing *Anopheles* mosquitoes to antimalarials blocks *Plasmodium* parasite transmission. Nature 567: 239–243. 30814727 10.1038/s41586-019-0973-1 PMC6438179

[2] WHO, 2018 World Malaria Report. http://www.who.int/malaria/media/world-malaria-report-2018/en/. Accessed November 19, 2018.

[3] Beier JC Muller GC Gu W Arheart KL Schlein Y, 2012 Attractive toxic sugar bait (ATSB) methods decimate populations of *Anopheles* malaria vectors in arid environments regardless of the local availability of favoured sugar-source blossoms. Malar J 11: 31. 22297155 10.1186/1475-2875-11-31 PMC3293779

[4] Anopheles gambiae 1000 Genomes Consortium, 2017 Genetic diversity of the African malaria vector *Anopheles gambiae*. Nature 552: 96–100. 29186111 10.1038/nature24995 PMC6026373

[5] Weedall GD 2019 A cytochrome P450 allele confers pyrethroid resistance on a major African malaria vector, reducing insecticide-treated bednet efficacy. Sci Transl Med 11: eaat7386. 30894503 10.1126/scitranslmed.aat7386

[6] Griffing SM Tauil PL Udhayakumar V Silva-Flannery L, 2015 A historical perspective on malaria control in Brazil. Mem Inst Oswaldo Cruz 110: 701–718. 26517649 10.1590/0074-02760150041 PMC4667572

[7] Blasco B Leroy D Fidock DA, 2017 Antimalarial drug resistance: linking *Plasmodium falciparum* parasite biology to the clinic. Nat Med 23: 917–928. 28777791 10.1038/nm.4381 PMC5747363

[8] Phillips MA Burrows JN Manyando C van Huijsduijnen RH Van Voorhis WC Wells TNC, 2017 Malaria. Nat Rev Dis Primers 3: 17050. 28770814 10.1038/nrdp.2017.50

[9] Fowler RE Billingsley PF Pudney M Sinden RE, 1994 Inhibitory action of the anti-malarial compound atovaquone (566C80) against *Plasmodium berghei* ANKA in the mosquito, *Anopheles stephensi*. Parasitology 108: 383–388. 8008451 10.1017/s0031182000075922

[10] Frampton JE, 2018 Tafenoquine: first global approval. Drugs 78: 1517–1523. 30229442 10.1007/s40265-018-0979-2

[11] Ranson H, 2017 Current and future prospects for preventing malaria transmission via the use of insecticides. Cold Spring Harb Perspect Med 7: a026823. 28507193 10.1101/cshperspect.a026823 PMC5666625

[12] Ahmed AM Maingon RD Taylor PJ Hurd H 1999 The effects of infection with *Plasmodium yoelii nigeriensis* on the reproductive fitness of the mosquito *Anopheles gambiae*. Inverterbrate Reprod Dev, 36, 217–222.

[13] Benelli G Beier JC, 2017 Current vector control challenges in the fight against malaria. Acta Trop 174: 91–96. 28684267 10.1016/j.actatropica.2017.06.028

[14] Zhu L 2015 Modelling optimum use of attractive toxic sugar bait stations for effective malaria vector control in Africa. Malar J 14: 492. 26643110 10.1186/s12936-015-1012-9 PMC4672472

[15] Burrows J Slater H Macintyre F Rees S Thomas A Okumu F Hooft van Huijsduijnen R Duparc S Wells TNC, 2018 A discovery and development roadmap for new endectocidal transmission-blocking agents in malaria. Malar J 17: 462. 30526594 10.1186/s12936-018-2598-5 PMC6287360

[16] Lucet IS Tobin A Drewry D Wilks AF Doerig C, 2012 *Plasmodium* kinases as targets for new-generation antimalarials. Future Med Chem 4: 2295–2310. 23234552 10.4155/fmc.12.183

[17] Ruecker A Mathias DK Straschil U Churcher TS Dinglasan RR Leroy D Sinden RE Delves MJ, 2014 A male and female gametocyte functional viability assay to identify biologically relevant malaria transmission-blocking drugs. Antimicrob Agents Chemother 58: 7292–7302. 25267664 10.1128/AAC.03666-14 PMC4249523

[18] Delves MJ Ramakirshnan C Blagborough AM Leroy D Wells TN Sinden RE, 2012 A high-throughput assay for the identification of malarial transmission-blocking drugs and vaccines. Int J Parasitol, 42: 999–1006. 23023046 10.1016/j.ijpara.2012.08.009

[19] Hemingway J, 2019 Battling disease by giving mosquitoes an antimalarial drug. Nature 567: 185–186. 30850737 10.1038/d41586-019-00648-2

